# Diagnostic applications and therapeutic option of Cascade CRISPR/Cas in the modulation of miRNA in diverse cancers: promises and obstacles

**DOI:** 10.1007/s00432-023-04747-6

**Published:** 2023-05-24

**Authors:** Tahereh Alinejad, Shabnam Modarressi, Zahra Sadri, Zuo Hao, Cheng Shui Chen

**Affiliations:** 1 The Key Laboratory of Interventional Pulmonology of Zhejiang Province, Department of Pulmonary and Critical Care Medicine, The First Affiliated Hospital of Wenzhou Medical University, South Baixiang, Ouhai District, Wenzhou, 325015 Zhejiang People’s Republic of China; 2 Department of Food Microbiology, Faculty of Science, University of Copenhagen, 1958 Frederiksberg C. Copenhagen, Denmark; 3 The Department of Biological Science, Molecular and Cell Biology, Dedman College of Humanities and Sciences Southern Methodist University (SMU), Dallas, TX USA

**Keywords:** CRISPR/Cas, miRNA, Cancer therapy, CRISPR–Cas9 library screen, Diagnostic

## Abstract

The Clustered Regularly Interspaced Short Palindromic Repeats (CRISPR)/Cas technology is a molecular tool specific to sequences for engineering genomes. Among diverse clusters of Cas proteins, the class 2/type II CRISPR/Cas9 system, despite several challenges, such as off-target effects, editing efficiency, and efficient delivery, has shown great promise for driver gene mutation discovery, high-throughput gene screening, epigenetic modulation, nucleic acid detection, disease modeling, and more importantly for therapeutic purposes. CRISPR-based clinical and experimental methods have applications across a wide range of areas, especially for cancer research and, possibly, anticancer therapy. On the other hand, given the influential role of microRNAs (miRNAs) in the regulations of cellular division, carcinogenicity, tumorigenesis, migration/invasion, and angiogenesis in diverse normal and pathogenic cellular processes, in different stages of cancer, miRNAs are either oncogenes or tumor suppressors, according to what type of cancer they are involved in. Hence, these noncoding RNA molecules are conceivable biomarkers for diagnosis and therapeutic targets. Moreover, they are suggested to be adequate predictors for cancer prediction. Conclusive evidence proves that CRISPR/Cas system can be applied to target small non-coding RNAs. However, the majority of studies have highlighted the application of the CRISPR/Cas system for targeting protein-coding regions. In this review, we specifically discuss diverse applications of CRISPR-based tools for probing miRNA gene function and miRNA-based therapeutic involvement in different types of cancers.

## CRISPR system and mechanism

In 1987, scientists discovered a CRISPR locus in the bacteria *Escherichia coli*. This locus was located near the 3′-end of the *iap* gene and was made up of five highly similar sequences of 29 nucleotides, arranged in a pattern of direct repeats separated by 32 nucleotides. At the time, researchers did not understand the significance of this discovery. Later, scientists found that many other short sequences repeats (SSRs) existed within this class of short sequences in archaea and bacteria, but not in eukaryotes or viruses (McGinn and Marraffini 2019; Jackson et al. 2017). There are two distinctive characteristics of interspaced SSRs: their repeat units (21–37 bp) are spaced by identically sized non-repetitive structural elements, and they are clustered in one or several locations on a chromosome. This distinct class of interspaced SSRs was first named—CRISPR in 2002 by McGinn and Marraffini (2019).

In 2005, for the first time, Bolotin et al. (2005) reported that invaders (extrachromosomal elements) from which CRISPR's spacer was derived were unable to infect the spacer-carrying strain. As a defense against bacteriophages, 40% of sequenced bacterial species and most archaea consist of clustered short palindromic repeats that are regularly spaced apart, which act in concert with CRISPR-associated (Cas) genes. Unlike other genetic engineering methods, by inserting small fragments of foreign nucleic acids between the repeats of the CRISPR locus, CRISPR–Cas systems can profoundly alter the host genome. This process is known as spacer acquisition (McGinn and Marraffini 2019; Jackson, et al. 2017).

Adaptation, expression, and interference constitute the three main stages in the immune response triggered by CRISPR–Cas. Protospacer-adjacent motif (PAM) recognition allows Cas proteins to connect to target DNA during the adaptation stage and cleave out protospacers, a section of the target DNA. In CRISPR arrays, the adaptation complex inserts the protospacer DNA into the array as a spacer after duplicating the repeat at the 5ʹ-end. It has been established that certain CRISPR–Cas systems employ an alternative mechanism for adaptation, specifically through the acquisition of spacers via reverse transcription of RNA by an enzyme encoded at the CRISPR–Cas locus (Makarova et al. 2020b; Gilbert et al. 2013). At the expression stage of the CRISPR array, pre-CRISPR RNA (pre-crRNA), a single transcript that is converted into mature CRISPR RNAs (crRNAs), each of which contains the spacer sequence and sections of the flanking repeats, is typically created. Pre-crRNA processing in various CRISPR–Cas variations is carried out by a unique component of a multiprotein Cas complex, a single Cas protein with several domains, or non-Cas host RNases (Steens et al. 2021). The protospacer (or a similarly related region) in the invading genome of a virus or plasmid is recognized by a Cas nuclease (or nucleases), which either exists in the effector or is recruited at the interference stage and is then cut and rendered inactive. The crRNA normally serves as a cue for this recognition and is frequently linked to the processing complex (protein) (Pinilla-Redondo et al. 2020; Yan et al. 2019).

## Diversity, modularity, and efficacy of CRISPR–Cas

Genome editing is a rapidly advancing field in biotechnology that allows for precise and targeted manipulation of DNA sequences. Among the various genome editing technologies developed in recent years, such as zinc finger nucleases (ZFNs) and transcription activator-like effector nucleases (TALENs), CRISPR–Cas system stands out for its ease of use, efficiency, and versatility. The CRISPR–Cas system has been commonly employed as a versatile tool for genome engineering since the 2012–2013 period (Wang and Li 2021). A detailed examination of the available genomes permitted the classification of the diverse Cas clusters into two classes, six types (I to VI), and 33 subtypes (Makarova et al. 2020a). Type I, III, and IV are comprised in Class 1 systems, whereas types II, V, and VI are included in Class 2 systems. These six categories have the following targets: types I, II, and V target DNA; types VI targets RNA, and type III system targets both RNA and DNA (Chen et al. 2016). To conduct interference, class 1 and class 2 systems act differently. For example, the class 1 system demands distinct combinations of Cas proteins, such as Cas3 (sometimes linked to Cas2), Cas5–Cas8, Cas10, and Cas11; however, class 2 systems require a large diversified proteins, such as Cas9, Cas12, and Cas13 in type II, type V and type VI systems respectively (O’Connell 2019). The type I system encloses Cas3 and the RAMP superfamily (which includes Cas5 and Cas6), while the bacterial type II system includes Cas9 and type III Cas10 found in bacteria and archaea (Liu et al. 2018). A CRISPR RNA (crRNA) guide acts jointly with a trans-activating CRISPR RNA (tracrRNA) scaffold in type II CRISPR–Cas systems aiming at organizing Cas9 to identify and cleave foreign DNA targets (Strecker et al. 2019). Each of the six types of CRISPR–Cas systems has distinct characteristics and functions: Type I is characterized by the presence of multiple CRISPR-associated (Cas) genes, including Cas1 and Cas2, that are involved in the acquisition and integration of new spacer sequences into the CRISPR array. The interference mechanism of Type I systems is mediated by multi-subunit ribonucleoprotein complexes that degrade invading nucleic acids (Makarova et al. 2020b). Type II is identified by the presence of a single Cas gene, Cas9, and a small RNA molecule that guides the system to specific sequences in the invading nucleic acid. Type II systems use the Cas9 protein to introduce double-stranded breaks in the target DNA (Gilbert et al. 2013). Type III systems are characterized by the presence of multiple Cas genes, including Cas10, Cas11, and Cas12, which are involved in the interference mechanism. The mechanism of Type III systems is mediated by crRNA-activating complexes that degrade invading nucleic acids (Steens et al. 2021). Type IV systems are characterized by the presence of multiple Cas genes, including Cas12 and Cas13, which are involved in the interference mechanism. The mechanism of Type IV systems is mediated by crRNA-activating complexes that degrade invading nucleic acids (Pinilla-Redondo et al. 2020). Type V systems are characterized by the presence of multiple Cas genes, including Cas14 and Cas12, which are involved in the interference mechanism. The mechanism of Type V systems is mediated by crRNA-activating complexes that degrade invading nucleic acids (Yan et al. 2019). Type VI CRISPR–Cas systems are unique in that they have a single Cas protein, Cas13, that is responsible for both recognizing and cleaving the target RNA. This differs from other CRISPR–Cas systems, which typically use multiple Cas proteins for these tasks (O’Connell 2019). The CRISPR–Cas9 system’s structural changes could be used in a variety of fields other than genome editing, such as the discovery of driver gene mutations and high-throughput gene screening, epigenetic modulation to reveal functional insights about chromatin modification, gene regulation, and genome organization, nucleic acid detection to aid clinical diagnosis and distinguish between different pathogen strains, and transcriptional regulation by targeted gene inhibition/interference (CRISPRi), and activation (CRISPRa); also, a direct conversion of C·G to T·A or A·T to G·C without double-stranded DNA breaks (DSBs) is made possible by genomic base editing, in which the Cas enzyme is coupled with naturally occurring or artificial deaminases. Platforms for genome editing have strong implications for cancer research and maybe anticancer therapy (Chen et al. 2016; Liu et al. 2018).

The Class 2/Type II CRISPR/Cas9 system is a powerful tool that has been developed to edit, regulate, and visualize the genomes of various organisms, particularly the yeast *Saccharomyces cerevisiae* (Slaymaker et al. 2016; Zetsche et al. 2017). At the core of this method is the identification and cleavage of RNA from invasive viral and plasmid DNA. Different species of Cas9 proteins have varying sizes and cleavage characteristics. The two main families of class 2 nucleases that have been used for genome editing in human cells are Cas9 and Cas12a. Cas9 is a dual-RNA-guided nuclease that requires both crRNA and tracrRNA and contains both HNH and RuvC nuclease domains. On the other hand, Cas12a is a single-RNA-guided nuclease that only requires crRNA and contains a single RuvC domain (Strecker et al. 2019). To achieve specific DNA targeting, crRNA can be combined with transactivating crRNA to produce a two-component RNA structure, directing Cas9 to specific DNA sequences with the help of tracrRNA. Another option is to create a single-guide RNA (sgRNA) by combining crRNA with tracrRNA. Cas9 recognizes the target sequences and the nearby PAM, causing site-specific DSBs. The cell must then repair the break through non-homologous end joining and/or homologous recombination to survive (Sander and Joung 2014).

Hopefully, genetic disorders will one day be treated with CRISPR–Cas9. To improve the CRISPR–Cas9’s application, some modifications and consideration are required: Cas9 ribonucleoprotein (RNP) complexes that have already been put together are injected into the target tissue or circulation act as a potential technique for delivering Cas9 into individuals or living things. For this method to be effective, and to withstand proteases and RNases in the blood or target tissue, Cas9 must be sufficiently stable (Harrington et al. 2017). One of the challenges of using CRISPR–Cas for therapeutic applications is the short lifespan of the Cas9 protein, which can limit the duration and effectiveness of the treatment. To overcome this limitation, several strategies have been proposed to extend the lifespan of the Cas9 protein in cells. One strategy is to use modified forms of the Cas9 protein that have been engineered to have increased stability. For example, a mutant version of Cas9 called eSpCas9 (enhanced Staphylococcus aureus Cas9) has been shown to have improved stability and a longer lifespan in cells compared to wild-type Cas9 (Slaymaker et al. 2016). This variant has been engineered to have a longer half-life by introducing mutations in the HNH and RuvC domains of the protein. eSpCas9 has a half-life of up to 4 h in human cells, compared to 1.5 h for wild-type SpCas9. A SpCas9 variant with a D10A mutation in the RuvC domain shows an increase of the half-life of the protein up to 4 h 30 min in human cells (Slaymaker et al. 2016) CRISPR-Cpf1 has been shown to have a longer half-life than Cas9 is CRIS.PR-Cpf1. The Cpf1 protein is derived from the *Lachnospiraceae* bacterium, and it has been found to have a longer half-life than Cas9. For example, one study found that the half-life of Cpf1 in human cells was approximately 6 h, compared to 2 h for SpCas. Cpf1 also has a smaller guide RNA, which allows for greater specificity and reduces off-target effects. It also has a different cutting mechanism, resulting in more precise cuts (Zetsche et al. 2017). Another approach is to use a "dead" version of Cas9 protein called dCas9 that can still bind to specific DNA sequences but lacks nuclease activity. dCas9 can be used to regulate gene expression and control specific genes, and so it does not need to be long-lived (Li et al. 2019) Another solution is to use Cas9 protein that has been fused with intra-bodies, which are small peptides that specifically bind to Cas9, thereby extending its lifespan (Marschall et al. 2015) Efficient delivery methods that allow for sustained release of the enzyme are also beneficial to overcome the mentioned challenges. One example is the use of nanoparticle-based delivery systems, which have been shown to effectively deliver Cas9 to cells and prolong the activity of the enzyme. A study has shown that by encapsulating Cas9 in a polymeric micelle, the half-life of the protein increased from 1 h 30 min to 6 h in human cells (Zhang et al. 2021).

Although CRISPR–Cas9 is a powerful tool for genome editing, it can also result in off-target mutations, which are unintended changes to DNA at locations that are not the intended target. These off-target mutations can occur due to errors in the guide RNA (gRNA) that directs the Cas9 enzyme to the target site or by other gRNA-independent mechanisms. This is a significant concern, particularly for therapeutic applications of CRISPR–Cas9. To address this problem, researchers have developed two main approaches: off-target detection techniques and engineering the CRISPR system for higher accuracy. The development of these methods is crucial for improving the specificity of the CRISPR–Cas9 system and reducing off-target mutations (Zhang et al. 2021). One method to use is the Cas9 nickase, which has single-strand nuclease activity instead of double-strand, thus reducing the chance of cutting both strands of DNA at the wrong location.

Another approach is to engineer Cas9 protein mutations that guide the enzyme to the correct location more accurately. Another method is to use a variant of Cas9 called deactivated Cas9 (dCas9), which has been engineered to only bind to a specific DNA sequence (Martinez-Lage et al. 2020; Hsu et al. 2014). The other approach to reduce the occurrence of off-target mutations is to fuse Cas9 with a programmable DNA-binding domain, which improves the enzyme’s ability to target specific DNA sequences. Moreover, truncated gRNAs, which are shorter versions of the gRNA, direct the Cas9 enzyme to the target site. Class 2 types V-A CRISPR–Cas12a, which have additional features, such as shorter crRNAs and RNase activity for pre-crRNA maturation, have been used to improve the specificity of the CRISPR–Cas9 system. These features enable multiplex gene editing, which is the ability to edit multiple genes at the same time. Additionally, the DNase-dead Cas12a (ddCas12a) can be used as a platform for programming various forms of transcriptional regulation or epigenetic alteration of the genome without altering the genome's sequence (Lee et al. 2018).

The type-V CRISPR effector Cas12b (formerly known as C2c1) has been challenging to develop for genome editing in human cells, at least in part due to the high-temperature requirement of the described family members. The Cas12b protein is a dual-RNA-guided nuclease that needs both crRNA and tracrRNAs, and it is preferentially nicked by wild-type BhCas12b at 37 °C. Even though Cas12b proteins are frequently smaller than Cas9 and Cas12a, and thus attractive from the standpoint of intracellular delivery via viral vectors, the best-characterized Cas12b nuclease from *Alicyclobacillus acidoterrestris* (AacCas12b) exhibits optimal DNA cleavage activity at 48 °C, precluding its use in mammalian cells (Li et al. 2019; Wen et al. 2020).

## CRISPR and cancer—advancements and hopes

Since the first documented cancer treatment in Ancient Egypt, which involved the surgical removal of tumors, human beings have tried to overcome cancer. While surgery is still the primary therapy for cancer in the modern-day, it has its limitations due to tumor accessibility and location and does not prevent systemic malignancies. Classic cancer treatments with significant DNA damage and cell death started to be made available in the twentieth century. Currently, the majority of cancer treatments utilized in clinical settings are conventional therapies, such as radiotherapy and chemotherapy. However, it has been observed that not all cancer types are responsive to these treatments. This has led to an increased focus on the development and investigation of novel therapeutic strategies for cancer treatment. Clinicians currently employ a variety of methods for treating cancer: surgery, radiotherapy, chemotherapy, targeted therapy, and immunotherapy. Despite improvements in therapeutic strategies, cancer still threatens more than nine million lives annually (www.clinicaltrials.gov). There is undoubtedly still a lot to learn about the biological activity of cancer cells and the molecular causes of disease. Cancer development includes many genetic abnormalities that can deregulate several genes, despite other hereditary illnesses like Duchenne muscular dystrophy or cystic fibrosis (Martinez-Lage et al. 2020; Hsu et al. 2014). In the context of cancer gene therapy, targeting a single gene is typically insufficient to destroy cancer cells, yet many malignancies depend on the presence of a single oncogenic event that can reorganize cells by altering downstream molecular and (epi)-genetic pathways and result in tumorigenesis (Martinez-Lage et al. 2020). Genome editing using CRISPR/Cas9 technology is a promising platform to cure cancer; however, there are still certain technical hurdles to overcome, including (i) targeting efficacy, (ii) off-target consequences, and (iii) in vivo delivery. The effectiveness of this method is further increased by strategies that combine gene editing with other treatment modalities (such as chemotherapy), enabling better outcomes with fewer chemotherapy dosages for the destruction of cancer cells.

Over 600 somatic mutations that cause cancer have been discovered through collective global effort and are listed online (COSMIC v82 database). In the 1980s, mutated RAS genes, among the earliest oncogenes, were first found in human tumors (Cox et al. 2014). Cancers, such as pancreatic ductal adenocarcinomas (PDACs), colorectal adenocarcinomas (CRCs), lung adenocarcinomas, and gastric adenocarcinomas, are all caused by oncogenic RAS mutations. About 25% of human malignancies have mutant RAS genes, which comprise the most frequently altered oncogene family. 85% of all RAS family oncogenic mutations are KRAS mutations (Lee et al. 2018; Wen et al. 2020).

In the field of cancer research, CRISPR/Cas9 technology is commonly employed for genomic editing, specifically for in vivo somatic gene fusion engineering in model organisms, such as *C*. *elegans*, flies, and mice. Additionally, CRISPR is increasingly being utilized as a screening technology. Consequently, CRISPR has rapidly evolved into a versatile toolset that facilitates the efficient generation of genetic variations across the entire genome. It is important to note that the application of CRISPR screening technology extends beyond cancer research and encompasses a broad range of fields, including clinical drug analysis and upstream biological discoveries (Patutina et al. 2018).

Distinguishing changes that are causing tumor evolution from passenger mutations is a difficult task in comprehending the cancer genome. The development of primary tumor growth and metastasis are separate yet related processes. The likelihood of finding metastases in a patient correlates favorably with the size of the main tumor, according to observations made in the clinic. Several alternative hypotheses have been proposed to explain the mechanism of metastasis in cancer. These include the possibility that larger tumors may disproportionately release more cells into the bloodstream, which subsequently colonize other sites, or that cells with high proliferative capacity may also possess a heightened ability for metastasis. It is also suggested that metastatic properties may only be acquired in advanced-stage tumors. Genetic screens are widely regarded as a recommended approach for evaluating phenotypes and identifying causative genes associated with various hallmarks of cancer progression. In a number of mouse models of oncogenesis, RNAi and the overexpression of open reading frames (ORFs) have been used to screen cancer-causing genes. Genes implicated in drug resistance and those required for cell viability have been found in several cell lines by Cas9-based screening. Accordingly, a genome-wide CRISPR/Cas9-mediated loss-of-function screen for tumor growth and metastasis is described by Chen et al. (2016) and Cox et al. (2014). Their research shows Cas9-based screening to be a reliable technique for in vivo gene phenotyping analysis in the context of cancer evolution.

Unlocking the molecular mechanisms of protein diversity and variation has therapeutic potential for cancer. In this respect, RNA editing makes early-stage tumor recognition possible, which is relevant to the process of an early cancer diagnosis. A potential RNA-editing tool with vast applications in science, medicine, and biotechnology is presented by RNA Editing for Programmable A to I Replacement (REPAIR). REPAIR technology can be used to edit transcripts with pathogenic mutations without requiring any specific sequence requirements. The RNA-guided, RNA-targeting type VI-B CRISPR effector Cas13b can efficiently and precisely knock down RNA, laying the groundwork for more effective tools for examining crucial genes and noncoding RNA as well as regulating cellular functions at the transcript level. The programmable single-effector RNA-guided ribonuclease Cas13 is present in Type VI CRISPR–Cas systems (Cox et al. 2014).

A CRISPR–Cas enzyme called Cas13a (formerly known as C2c2), which targets RNA in class 2 type VI, can be engineered to knock down and bind mammalian cell RNA. A preliminary screening of orthologues led to identifying Cas13a (LwaCas13a) from *Leptotrichia wadei* as the most effective interference assay in *E*. *coli* (Xue et al. 2016). In both mammalian and plant cells,* Leptotrichia wadei* (LwaCas13a) can be heterologously expressed for targeted knockdown of either reporter or endogenous transcripts. In terms of knockdown efficiency, LwaCas13a is comparable to RNAi, but with significantly reduced off-target effects, making it more suitable for therapeutic application. The next advantage is its ability to mediate nuclear RNA knockdown and multiplex knockdown (Xue et al. 2016). The detection of microRNA (miRNA) can be achieved through the utilization of the targeted RNA activity of Cas13a/C2c2. Cas13b, a newly discovered CRISPR–Cas effector, holds similar potential as previously characterized classes 2, such as Cas9 and Cpf1. The ability of Cas13b to process its own CRISPR array may also enable multiplex transcriptome engineering. Additionally, the incorporation of stem–loops similar to the Cas9-SAM system into VI-B functional long direct repeats may be possible. Both Cas13a and Cas13b exhibit potential for complementary applications in research and technology, similar to the utilization of Cas9 and Cpf1 (Nguyen and Chang 2018).

## CRISPR/Cas: a practical technique for examining and regulating miRNAs gene function

There have been very few studies using the CRISPR/Cas9 system to target non-coding RNA regions, including miRNAs for genomic depletion, as the vast majority of these studies focus on protein-coding genes (Hannafon et al. 2019). miRNAs are endogenous small RNAs that do not have coding functions. These 18–24 nucleotides regulate the translation or degradation of protein-coding genes post-transcriptionally by imperfect binding to 3′-untranslated regions (3′UTR) of their target mRNAs. A miRNA primarily functions by attaching to the 3′UTR of the target mRNA and decreasing the stability and translation efficiency of the target. By interacting with promoters, miRNAs are also known to influence gene expression in the nucleus (Jalali et al. 2022).

Considering their broad regulatory role and their diverse target mRNAs, miRNAs can regulate multiple normal and pathogenic cellular processes, such as cell cycle, tumorigenesis, migration and invasion, and angiogenesis, therefore acting as tumor suppressors or oncogenes (Hannafon et al. 2019; Taft et al. 2010). Cancer-associated miRNAs are diverse and distinct for various tissues and cancer types, indicating that they may be viable biomarkers for diagnostic and therapeutic targets. They are also said to be effective indicators of the prognosis for cancer (Oliveto et al. 2017). The expression of their proto-oncogenic and oncosuppressor mRNAs is regulated by autocrine and paracrine mechanisms involving both intracellular and extracellular miRNAs, which either causes the cells to undergo malignant transformation or maintains their normal homeostasis (Finn and and Searles 2012; Dalmay and Edwards 2006; Patutina et al. 2018). Upregulated oncogenic miRNAs (oncomiRNAs) or downregulated tumor-suppressive miRNAs are examples of miRNAs whose balanced expression is defective during carcinogenesis. For example, miR-21 is overexpressed in several types of cancer, including breast, lung, and ovarian cancer, and its high expression is associated with poor prognosis and resistance to chemotherapy. Inhibiting miR-21 with anti-miR-21 oligonucleotides has been shown to inhibit the growth of cancer cells in vitro and in vivo. Similarly, miR-155 is overexpressed in several types of cancer, including lymphoma and leukemia, and its high expression is associated with poor prognosis and resistance to chemotherapy. Inhibiting miR-155 with anti-miR-155 oligonucleotides has been shown to inhibit the growth of cancer cells in vitro and in vivo (Lu et al. 2005; Xue et al. 2016).

The development of several methods for exploring miRNAs and examining their roles in cell culture and animal models has been sped up as a result of these important miRNAs. miRNAs as targets for gene-targeted therapies to inhibit tumor growth has altered the paradigm, which until recently primarily focused on the down-regulation of coding mRNAs (Patutina et al. 2018; Nguyen and Chang 2018).

Inhibiting oncogenic miRNAs has been approached in a variety of ways in recent years. More than half of the protein-coding genes in mammals are projected to be negatively regulated by miRNA, which are found throughout the eukaryotic genome. Most biological processes, including apoptosis, differentiation, and proliferation, depend on miRNAs, and their transcription is carefully regulated (Hannafon et al. 2019; Lin et al. 2020; Chen et al. 2018). miRNA clusters frequently experience a sequence of duplication events that result in the creation of paralogues, which are believed to regulate a comparable set of genes and have similar biological properties. It has been claimed that the development of structural clusters will increase stability by delaying rapid degradation and assuring the synthesis of miRNAs that regulate a comparable group of genes, which will directly affect their activity.

The microprocessor complex, which is made up of the RNAase III enzyme Drosha and the double-stranded RNA-binding protein DGCR, processes miRNA genes into main miRNAs in the nucleus and subsequently precursor miRNAs (pre-miRNAs) in the cytoplasm. The RNA-induced silencing complex (RISC), which contains the RNAase III enzyme Dicer, Ago2, and other RNA-binding proteins, processes mature miRNA in the cytoplasm. miRNAs work through complementarity in their seed sequences, which bind to the 3′ 3′UTR of their target genes. The expression of miRNAs is dysregulated in several human illnesses, including cancer and cardiovascular conditions (Finn et al. 2018).

One miRNA can regulate hundreds to thousands of target genes, according to bioinformatics analyses based on the “seed sequence matching” theory; however, many miRNAs can also activate the same gene. The most popular approaches used in cell and molecular biology to investigate a target gene are gain and loss-of-function. The main loss-of-function techniques used in miRNA research are genetic knockout, miRNA sponges, and antisense inhibitors that target specific miRNAs (Yang et al. 2013; Sun et al. 2017). The nature of miRNA, which is a transcript from DNA, as well as synthetic mimics or expression vectors to increase the abundance of miRNA in cells, are widely used in the most fundamental and translational studies (Graham and Hart 2021). However, in comparison to the efficacy of overexpression strategies, the developed methodologies in the downregulation of miRNA are relatively less reliable. This is partly because miRNAs are just 22 nt long, which makes them more resistant to the cleavage caused by other tiny molecules like siRNA (Yang et al. 2013).

To better understand miRNA function and to treat disorders connected to these functions, miRNA inhibition is used. Complementary antisense oligonucleotides (ASOs) that have been chemically altered are currently employed extensively in vitro as miRNA silencers, although ASOs typically need expensive modifications and frequently have off-target effects (Zhao et al. 2014).

It is technically challenging to produce loss-of-function miRNA mutations via homologous recombination since the transcripts are often brief. Therapeutic approaches targeting miRNA expression have been proposed as a way to treat cancer. One such approach is miRNA gene editing using CRISPR–Cas, which can be used to either increase or decrease miRNA expression in cancer cells. Increasing miRNA expression in cancer cells can be used as a means of restoring normal gene regulation and suppressing the growth and proliferation of cancer cells. This approach has been shown to be effective in preclinical studies of various types of cancer, including lung cancer and glioblastoma (Tian et al. 2017). On the other hand, decreasing miRNA expression in cancer cells can be used to disrupt the tumor-promoting effects of oncogenic miRNAs, which are miRNAs that promote the growth of cancer cells. This approach has also been shown to be effective in preclinical studies of various types of cancer, including breast cancer and leukemia (Yang et al. 2018; Wallace et al. 2016). Cas9 can be guided to a sequence of interest using a specially created crRNA–tracrRNA duplex, known as a “gRNA”, and produce DSBs that silence genes (Ryu et al. 2020; Hazafa et al. 2020). The CRISPR/CRISPRi system can be easily adapted to target a specific miRNA sequence by replacing the crRNA within a single repression vector, which only requires a change to a 20-base pair sequence. This feature simplifies the construction process of the CRISPR/CRISPRi system, particularly when creating vectors that target multiple miRNAs (Hazafa et al. 2020). The CRISPRi approach may also be advantageous since a single vector can less toxically silence a number of targets within a miRNA cluster. To identify certain target sequences, miRNAs employ partial base pairing, with the short seed sequence (the miRNA's nucleotides 2–8) playing a key role. This property makes it difficult to predict which genes are targeted by a particular miRNA while also allowing one miRNA to target tens or hundreds of genes. According to previous research by Broughton and his colleagues (Hoffmann et al. 2019), the global genome editing tool CRISPR could be modified to find miRNA targets with biological value thanks to its robust and consistent performance. First, it is simple to modify CRISPR knockout screens to find genes whose inactivation decreases mutant phenotypes of miRNA. Second, placing sites whose mutation reproduces the symptoms of miRNA mutants can be done using a custom strategy to target specific miRNA-binding sites via CRISPR. The functionality of individual miRNA-binding sites can be evaluated through the use of specialized sgRNA libraries. These tailored libraries can be introduced into wild-type cells, following which cells displaying binding site mutations that replicate specific characteristics of the miRNA mutant phenotype can be selectively chosen. Knockout screens are a mere demonstration of the potential of CRISPR as a screening platform. The utilization of CRISPR-based screens for systematic disruption has the potential to significantly expand the scope of questions that can be explored using functional genomics techniques (Bachmayr-Heyda et al. 2015).

Alternatively, the CRISPR system can knock out the microRNA gene cluster through genomic deletion or homologous recombination (HR); however, genomic deletion demand the construction of two sgRNAs, and HR necessitates the creation of a second donor vector (Luo et al. 2016). By altering both of Cas9's nuclease domains, a catalytically dead Cas9 (dCas9) is produced, extending the capabilities of the CRISPR toolkit for transcriptional regulation. The mutant can still be directed to specific genomic locations, but it is incapable of cleaving DNA. CRISPR interference (CRISPRi) is a technique that uses the derivative system to sterically occupy a gene’s promoter or gene body in order to prevent the recruitment of transcriptional machinery or the extension of transcription. Because only one sgRNA needs to be designed to target the gene cluster's promoter region using CRISPRi, inhibiting a microRNA gene cluster will be easier (Luo et al. 2016).

Strategies to restrict CRISPR–Cas9 activity to certain cells and organs are greatly desired when using CRISPR technology in vivo. Maximizing specificity and, thus, the safety of a medication in the context of therapeutic genome editing in human patients, is essential. Several different methods have been used for delivering the CRISPR/Cas9 components to target cells for gene editing and improving specificity (Table 1), including viral delivery through which the CRISPR/Cas9 components are packaged into a virus, such as a lentivirus or adenovirus, which is then used to infect the target cells. This method is widely used in research and has been shown to be efficient in editing genes in a variety of cell types. Advantages of using a virus to deliver CRISPR/Cas9 include the ability to infect both dividing and non-dividing cells, high transfection efficiency, and the ability to target specific cell types by engineering the virus to infect cells with specific surface markers. However, the use of viruses for gene editing in humans raises safety concerns, as well as ethical issues, and the potential for unintended effects on the host genome (Xu et al. 2019). Lentivirus-mediated delivery of CRISPR/Cas9 components has been used to edit the genomes of human T cells to treat cancer and to correct the genetic mutations that cause sickle cell anemia and beta-thalassemia. Adenovirus-mediated delivery of CRISPR/Cas9 components has been used to correct a genetic mutation that causes cystic fibrosis in airway epithelial cells (Liu et al. 2017). The other delivery method is lipid nanoparticle delivery. Lipid nanoparticles can be used to deliver CRISPR/Cas9 components into a wide range of cell types, including primary cells and stem cells, and they have been shown to be efficient and specific in gene editing. The CRISPR/Cas9 components are packaged into lipid nanoparticles, which can enter cells via endocytosis. Lipid nanoparticles are also non-toxic and non-immunogenic, making them a safer alternative to viral delivery (Finn et al. 2018). However, one of the major disadvantages of this method is that it has lower transfection efficiency compared to viral delivery and electroporation methods. They have been used to deliver CRISPR/Cas9 components to edit the HBB gene that causes beta-thalassemia in hematopoietic stem cells, as well as to correct a genetic mutation that causes Leber congenital amaurosis (LCA) in retinal pigment epithelial cells (Yang et al. 2013; Sun et al. 2017). Electroporation which is a brief electrical pulse has also been proven to be effective delivering strategy. It can be applied to the cells to create temporary pores in the cell membrane, through which the CRISPR/Cas9 components can enter. This method is fast, efficient, and can be used to deliver CRISPR/Cas9 components into a wide range of cell types, including primary cells and stem cells. It is also relatively inexpensive and can be performed in a laboratory setting (Marschall et al. 2015). However, electroporation can also cause damage to cells and may not be suitable for editing genes in certain cell types. Electroporation has been used to deliver CRISPR/Cas9 components to edit the HBB gene that causes beta thalassemia in hematopoietic stem cells, as well as to correct a genetic mutation that causes cystic fibrosis in airway epithelial cells (Graham and Hart 2021). The CRISPR/Cas9 components are injected directly into the nucleus of individual cells using a fine needle. This method that has been called microinjection is highly specific and can be used to edit the genomes of individual cells, which makes it useful for studying the effects of specific gene edits. However, it is a time-consuming and labor-intensive process and is not suitable for editing large numbers of cells (Lino et al. 2018). Ultrasound waves are used to create temporary pores in the cell membrane, through which the CRISPR/Cas9 components can enter. This method is based on the same principle as electroporation but uses ultrasound waves to create temporary pores in the cell membrane. It has been shown to be efficient in delivering CRISPR/Cas9 components into a wide range of cell types and has the potential to be used in vivo. However, it is a relatively new method and more research is needed to fully understand its advantages and disadvantages (Ryu et al. 2020). CRISPR/Cas9 components are packaged into nanoparticles such as gold nanoparticles or polymeric nanoparticles, these nanoparticles can help in targeted delivery of CRISPR/Cas9 into cells. This method has a high specificity, as nanoparticles can be engineered to target specific cells. Additionally, it can increase the stability of the Cas9 protein and gRNA, which increase the efficiency of gene editing. However, the transfection efficiency of nanoparticles is still lower than viral delivery and electroporation methods. Gold nanoparticles conjugated with CRISPR/Cas9 components have been used to deliver the components to cells and to achieve efficient gene editing in vitro and in vivo (Lino et al. 2018). RNA ribonucleoproteins are another means of delivering the CRISPR/Cas9 components that uses pre-assembled RNP complexes. RNPs have been shown to have high efficiency and specificity, and they are also easy to use. Additionally, RNPs are less likely to cause off-target effects than plasmid DNA. However, RNPs are more expensive than plasmid DNA, and the Cas9 protein may not be as stable as other methods (Zhang et al. 2021). Chemical transfection is also a method that uses chemicals, such as liposomes, polymers, and dendrimers, to deliver the CRISPR/Cas9 components. This method is relatively simple, and it can be used to deliver the components to a wide range of cell types. However, it is less efficient than other methods and can be toxic to cells. Additionally, the transfection reagents can add variability to the results (Yang et al. 2013). Liposomes, which are spherical structures made of a lipid bilayer, can be used to deliver CRISPR–Cas9 components into cells. Liposomes can be made to specifically target certain types of cells, such as cancer cells (Hazafa et al. 2020). Up till now, almost any method of effectively delivering CRISPR–Cas components in vivo is likely to affect a variety of cell types and tissues aside from the one that is actually of (therapeutic) relevance. Because of the limited specificity, there are significant chances of adverse effects from the treatment. Making the activation of the CRISPR components dependent on endogenous, cell-specific signals is one way to overcome this constraint and ensure that the genetic disruption is only produced in the target cell population and not in off-target cells. miRNAs, which are tiny, non-coding, regulatory RNAs involved in eukaryotic gene expression regulation, are one type of such signal. miRNAs identify sequence motifs found on mRNAs that are complementary to the miRNA sequence because they are a component of the RNA-induced silencing complex (RISC). The RISC normally then causes mRNA degradation, translation inhibition, or both, leading to a suppression of gene expression (Hoffmann et al. 2019).

**Table 1:** Advantages and disadvantages of CRISPR–Cas9 thechnology

Delivery method	Advantages	Disadvantages	Examples	References
Plasmid DNA	Easily manufactured and purified, low cost	May not efficiently enter cells, transient expression	Gene knock-in and knock-out in human cells, Targeted mutagenesis in zebrafish	Wang et al. (2013) Cong et al. (2013)
Viral vectors (e.g., adeno-associated virus, lentivirus)	Can efficiently deliver CRISPR components to a wide variety of cell types, allows for stable integration and long-term expression	May elicit immune response, insert at undesired locations in the genome and have regulatory and ethical concerns	Gene therapy for sickle cell anemia, Knock-out of tumor suppressor genes in cancer cells	Cartier et al. (2009) Maeder et al. (2013)
Lipid nanoparticles	Can efficiently deliver CRISPR components to a wide variety of cell types	May be toxic to cells and/or have reduced stability, limited to in vitro and ex vivo applications	Gene editing in human pluripotent stem cells	Finn et al. (2018)
Electroporation	Can be used for primary cells and hard-to-transfect cell lines, allows for transient expression	May cause damage to cells, have limited efficiency	Editing the *HBB* gene that causes beta-thalassemia in hematopoietic stem cells	Graham and Hart (2021)
Microinjection	Can deliver CRISPR–Cas9 complex to specific cells or regions, allows for targeted and localized editing	Limited to small number of cells and requires specialized equipment, may not achieve long-term expression	Targeted mutagenesis in zebrafish, Gene editing in specific regions of the mouse brain, altering the genes of *Cynomolgus* monkey embryos by injecting a single solution into one-cell-stage embryos, specifically targeting, and disrupting the Ppar-γ and Rag1 genes	Lino et al. (2018) Varshney et al. (2016)
Nanoparticles	Can deliver CRISPR–Cas9 complex to cells with high efficiency, allows for targeted and localized editing	May be toxic to cells and/or have reduced stability	Gene editing in human pluripotent stem cells, Knock-out of disease-causing genes in human primary cells	Liu et al. (2017) Nguyen et al. (2020)
RNA ribonucleoproteins (RNPs)	High efficiency, specificity, and stability	Limited delivery options and potential for off-target effects	Delivery of gRNA using a ribonuclease called T7 RNase, delivery of the Cas9 protein itself using an RNase called ribonuclease T2	Zhang et al. (2021) Endo et al. (2019)
Chemical transfection	High transfection efficiency and low toxicity	Limited stability of the delivered RNA	Using lipofection to deliver CRISPR–Cas9 components into lung cancer cells to study the role of a gene called *KRAS* in the development of lung cancer	Hazafa et al. (2020)

CircRNAs are thought to negatively control miRNAs in their putative role as miRNA sponges, significantly enhancing the competitive endogenous RNA (ceRNA) network. A study revealing a reduction in single-nucleotide polymorphisms at miRNA-binding sites in circRNAs provides additional support for the miRNA sponge function of circRNAs. However, it is currently unclear if all circRNAs function as miRNA sponges (Bachmayr-Heyda et al. 2015). The transport of miRNAs is another proposed use for circRNAs. Given the potential role of circRNAs as miRNA sponges, circRNAs might inhibit miRNA activity by binding freshly generated mature miRNAs. Since miRNAs are thought to only partially bind to the circRNA sponge, they do not cause the circRNA to be degraded by miRNAs. In order to control translation or transcription or to mediate transcript degradation, miRNAs would therefore be less disposable for binding transcripts or promoters (Bachmayr-Heyda et al. 2015).

As a result, the situation regarding epigenetic regulation by miRNAs would be more stable with more CirRNAs. In Hansen et al.'s study, it was speculated that at least ciRS-7 has postulated that cleavage of circRNAs is mediated by perfect miRNA binding, resulting in the release of all imperfectly bound miRNAs (Zhu et al. 2013). Since a small number of circRNA-specific miRNAs trigger the release of a large number of (other) miRNAs, which are subsequently active for regulation, this method appears to be quite effective. According to studies, circRNA expression was generally downregulated in ovarian and colorectal malignancies and was adversely connected with the severity and proliferation of the disease. The proposed mechanism of epigenetic regulation via miRNAs and the competing endogenous RNA network was validated using non-cancerous cell lines and normal human tissue in three separate contexts. If this mechanism is found to be a general principle in human cells, it has the potential to greatly expand our understanding of epigenetic regulation and its implications for various cancer entities and tissues. Results from cell lines must be translated with greater care to the in vivo setting, especially when the ceRNA network is implicated (Yang et al. 2012).

As with cancer, miRNAs play an essential role in initiating and developing cardiovascular diseases. Furthermore, it was discovered that circulating miRNA signatures were related to cardiovascular risk, and a brand-new method of intercellular communication was postulated (Ma et al. 2007; Plummer et al. 2013). miRNAs can be classified based on the mechanism of their regulation into two types: homo-clusters and hetero-clusters. Homo-clusters are composed of miRNAs from the same family and typically regulate their targets in a single step. Hetero-clusters, on the other hand, are composed of miRNAs from different families and typically regulate their targets in multiple steps, resulting in a delayed response (Zhang et al. 2017).

The roles of miRNAs in vitro and in vivo have been investigated using gain- and loss-of-function methods. miRNA expression has been silenced via a variety of loss-of-function techniques, such as plasmid- or viral-based antagomiRNA, miRNAoff and decoymiRNA, or synthetic antisense miRNA inhibitors. These techniques result in a partial silencing of miRNA expression. But CRISPR/Cas9 is a cutting-edge method for loss-of-function investigations because it mutates pre-miRNA sequences to disrupt miRNA expression and disrupt miRNA genes (Yang et al. 2013; Huo et al. 2017).

Malignant tumors have a terrible prognosis in large part because they can invade the human body and because standard treatments like surgery, radiotherapy, and chemotherapy cannot get rid of dispersed tumors. Neural stem cells (NSCs) have been developed and used as a novel gene delivery vector for targeted cancer gene therapy from bench to bedside over the past ten years (Luo et al. 2016; Yang et al. 2012). NSCs can move through the brain parenchyma to target tumor foci, including the primary tumor site and distant metastatic locations, when there are brain tumors present. According to reports, chemokines generated from hypoxic tumors via signaling pathways like SDF-1/CXCR4 and HGF/c-Met encourage this tumor-tropic activity. The tumor tropism of NSCs has been thoroughly investigated in animal tumor models for the targeted delivery of anticancer medicines to both primary tumor masses and distant tumor metastases (Luo et al. 2016; Zhu et al. 2013).

### Identification of microRNA as biomarkers through CRISPR technique

Numerous studies concentrating on the control mechanisms of miRNAs have been encouraged by the discovery that they are crucial in the origin and progression of many diseases. Additionally, miRNA profiling can be utilized for cancer detection and classification because it has been shown that the expression patterns of these miRNAs are associated with several clinical aspects of cancer. miRNAs have been found to be stable under various harsh conditions, including exposure to RNases, multiple freeze–thaw cycles, and extreme pH. This stability has led to the identification of circulating miRNAs as powerful biomarkers for point-of-care diagnosis of various disorders (Panwar et al. 2017).

Numerous studies have proposed a role for circulating miRNAs as novel cancer biomarkers due to their extraordinary stability in plasma and serum and the ability to measure their levels utilizing noninvasive techniques. One such indicator of tumor aggressiveness is miR-10b-5p. Other studies that showed increased levels of circulating miR-10b in patients with metastatic breast cancer and worsening clinical stage also lend weight to this assertion. Research on miR-10b has shown its importance in invasion and metastasis (Ma et al. 2007). Additionally, the vasculature in high-grade breast cancer has been found to be highly expressed by miR-10b (Plummer et al. 2013). Accordingly, tumor and microenvironment factors may directly contribute to the increased levels of miR-10b in the bloodstreams of advanced breast cancer patients.

High efficacy and low-cost miRNA detection may help with noninvasive diagnosis, prognosis, and other standard laboratory chores. The highly specific nucleic acid binding capabilities of the CRISPR–Cas systems suggest that novel strategies might be available. Based on these approaches, nucleotide detection techniques have been developed in recent studies. Nevertheless, these techniques were not suitable for detecting short miRNAs. Noticeably, it has been possible to introduce functional proteins, particularly enzymes and reporters, to specific genome loci for various applications, including “base editing”, imaging, and signaling using a protein-fusing method, by producing Cas9 mutants with inactivated nuclease activity, known as catalytically dCas9 (Zhang et al. 2017; Komor et al. 2017).

For the low-cost and simple detection of miRNAs with fM-level sensitivity and single-base specificity, Qiu et al. (2018) developed the innovative RCA-CRISPR-split-HRP (RCH) technique with synthetic bio components. In this procedure, miRNAs were isothermally amplified by RCA for the detection test and used for in vitro dCas9 binding and split HRP activity evaluation. The presence of particular miRNAs was identified by a light yellow to blue color change in the TMB substrate.

**Fig. 1:**
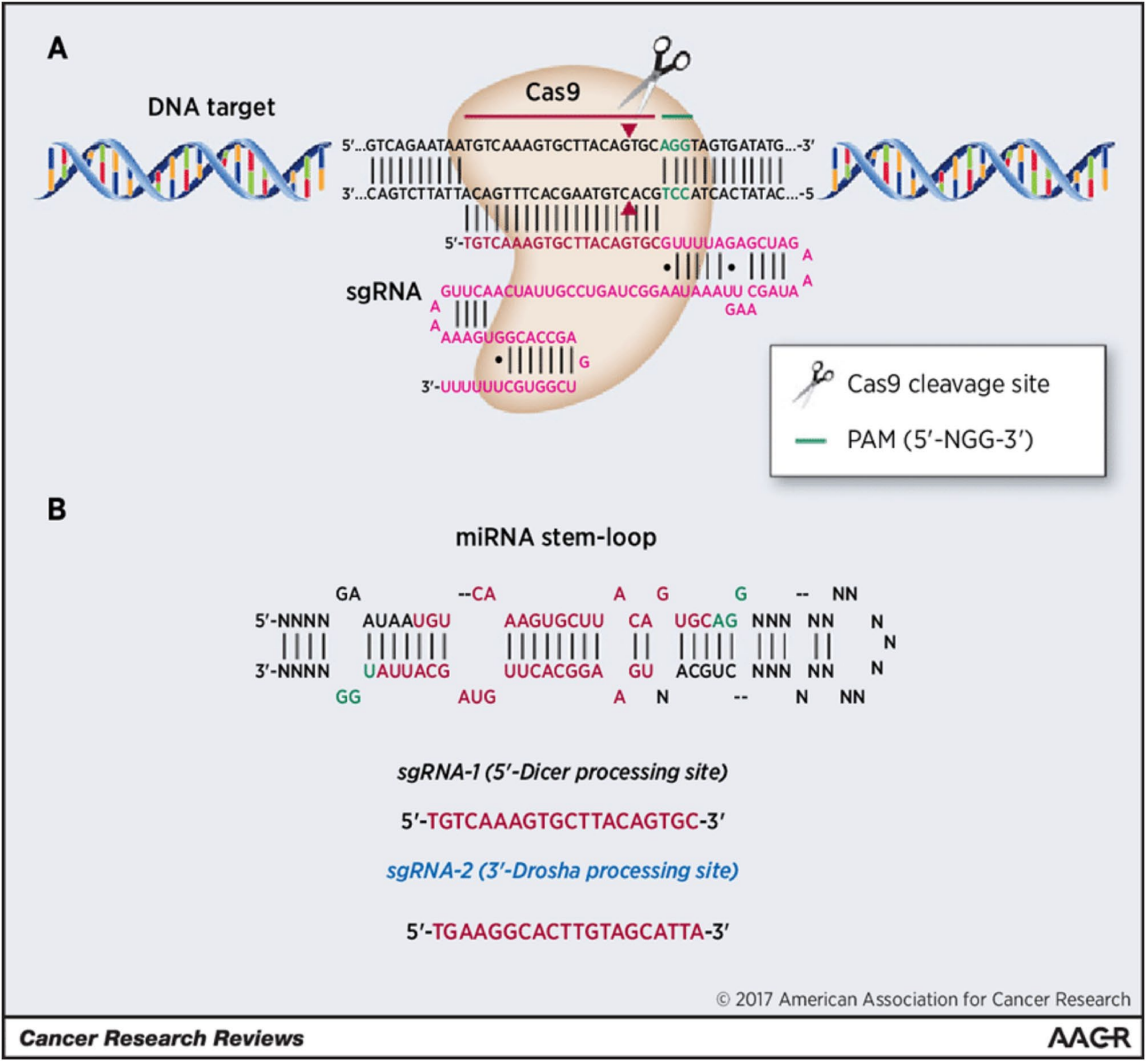
The method of CRISPR/Cas9-mediated miRNA editing is shown in a schematic diagram. TheCas9 nuclease from *S. pyogenesis* was directed to genomic DNA, such as thehsa-miR-17 gene, via a sgRNA made up of a scaffold and a 20-nt guide sequence (red) (magenta). The DNA target (red line on top strand) and the guide sequence pair upstream of the necessary 50-NGG neighboring motif (PAM; green). Around 3 kb upstream of the PAM, the Cas9 nuclease causes a double-strand break (DSB) in the targeted genomic DNA (such as the Drosha or Dicer-processing sites) (red triangles). DNA repair mechanisms mediated by the host are used to fix the DSB caused by Cas9 nucleotide; *bp* base pairs. **B** CRISPR/cas9 can change the miRNA biogenesis process. The production of mature miRNAs in cells can be triggered by SgRNAs that target the sequences within or nearby Drosha- and Dicer-processing sites, respectively, in the secondary stem–loop structure of primary miRNA sequences. Red is used to represent both of the mature miR-17 sequence’s arms in the hairpin's stem–loop. PAM sequences are highlighted in green. The production of mature miRNAs in cells can be triggered by SgRNAs that target the sequences within or nearby Drosha- and Dicer-processing sites, respectively, in the secondary stem–loop structure of primary miRNA sequences. Red is used to represent both of the mature miR-17 sequence's arms in the hairpin's stem–loop. PAM sequences are highlighted in green (Aquino-Jarquin 2017)

Another approach is CRISPR-Dx, which uses CRISPR–Cas12a to logically integrate DNA circuits in order to identify miRNAs in a sensitive and economical manner (Chen et al. 2018). In this technique, the CRISPR–Cas12a system is utilized for the detection of miRNA biomarkers through the conversion and amplification of each target into multiple programmable DNA duplexes. This is achieved through the use of a modular catalytic hairpin assembly (CHA) circuit. The integration of this method provides a general assay for the highly sensitive detection of miRNA biomarkers. This assay delivers sub-femtomolar sensitivity for several miRNA biomarkers simply by tweaking the variable areas in the CHA modules, which increases the detection limit of CRISPR-Dx in the analysis of miRNA by 3–4 orders of magnitude. Two significant engineering innovations are present in the proposed CRISPR-CHA. First, the seed sequence-dependent DNA targeting mechanism of CRISPR-12a is utilized to improve the collateral cleavage-mediated signal amplification by adding unpaired sites into the DNA duplex outputs. Second, a universal method for miRNA probing is made possible by the modular construction of CHA components, which enables the use of the same gRNA to probe the DNA duplex outputs from various miRNA targets. The adaptability of CRISPR-CHA has been established in the isothermal detection of miRNA biomarkers, such as miR-21, miR-141, and miR-155, with sub-femtomolar sensitivity, which is noticeably superior to that of Cas13a-based approaches (Hong et al. 2020; Wang and Wang 2020). Many tumor types, including brain, liver, prostate, and lung cancers, are thought to have an abnormal amount of miR-21, which is linked to their origin, progression, and prognosis (Chen et al. 2018). Fig. 1.

### miRNAs expression

#### Breast cancer

The most frequent type of cancer among women is breast cancer. Due to the establishment of screening programs and the creation of novel therapies, mortality rates have drastically decreased over the previous 20 years. Breast cancer is a molecularly diverse family of neoplastic illnesses, with significant interpatient variability in genetic alterations, cell makeup, transcriptional patterns, and response to therapy. As a result, there is a rising need for new diagnostic methods that attempt to molecularly annotate each patient's specific condition and create more specialized treatment plans.

Such inventive methods can be developed thanks to CRISPR-associated (Cas) technology. For instance, the RNA-specific C2c2 system can be employed in diagnostics to describe the mutational repertoire and transcriptional breast cancer markers by enabling ultrasensitive nucleic acid detection. CRISPR/Cas9 technology can be used to specifically alter oncogenes and tumor-suppressor genes implicated in disease pathogenesis for disease modeling. Gene therapy can be created using CRISPR/Cas9 in medical settings, and catalytically dCas9 can be utilized to change the epigenetic makeup of cancerous cells. CRISPR/Cas9 can modify immune cells to guide them against cancer cells and enhance antitumor immune responses as immunotherapy becomes more prevalent in cancer treatment. Even though there have been numerous discoveries that have helped us understand how breast cancer begins, grows, and progresses, we still only have a partial grasp of the molecular pathways that underlie breast cancer pathogenesis. It has been shown that in cancer, dysregulation of miRNA can change the synthesis of either an oncogenic protein or a tumor suppressor protein, which in turn promotes the growth and development of tumors.

It has been demonstrated that CRISPR/Cas9 technologies improve the effectiveness of modifying the differentiated genome in human cells. Accordingly, miR130a expression was downregulated using this method. The research of miR130a’s molecular significance in breast cancer may then be developed using this methodology. The expression of mature miR130a-3p and miR130a-5p was silenced in the MCF7 cell line using the CRISPRs method. The pSpCas9(BB)-2AGFP (PX458) plasmid was used to clone the 20 bp sequences that target the stem–loop, 3', and 5' ends of miR130a. The positive clones were validated by sequencing. Real-time quantitative PCR was used to confirm the suppression activity. The pre-miR130a stem–loop, 3p, or 5p was silenced in the cytoplasm using the CRISPR/Cas9 system. The RNAse III enzyme Dicer will break down premiR130a in the cytoplasm, producing mature miRNAs that are integrated into a multiprotein complex. This method may help to control the amount of miRNA in different cell types to provide biological answers to clinical concerns (Abdollah 2017).

The miR-23-27-24 family has two paralogs: the intragenic miR-23b cluster (miR-23b-27b-24-1) on chromosome 9 within the C9orf3 gene, and the miR-23a cluster (miR-23a-27a-24-2) on chromosome 19 (Hurst et al. 2009). Only one nucleotide at the 3′ end separates miR-23a and miR-27a from their paralogs, but despite this, different expression patterns are seen between these two clusters, and both are predicted to have largely identical targets. However, according to miRTarBase, less than 15% of the predicted targets for both miR-23a and miR-27a have been validated (Panwar et al. 2017; Chou et al. 2016). The most current research indicates that miR-23b and miR-27b have opposing effects on the growth of cancer. Expression of miR-23b has been demonstrated to affect chemoresistance and tumor cell dormancy, as well as to limit tumor growth, invasion, angiogenesis, and metastasis. In human gliomas, prostate, bladder, breast, and gastrointestinal malignancies, it is down-regulated (Hannafon et al. 2019).

By genetically removing these miRNAs via CRISPR/Cas9 editing, Hannafon et al. (2019) identified the precise function of miR23b and miR-27b in the advancement of breast cancer. Their research revealed that genetically depleting miR-23b and miR-27b suppresses tumor growth, proving that these miRNAs are in fact oncogenic in breast cancer. Additionally, they proposed that therapeutic targeting of these miRNAs is a feasible management strategy for breast cancer because dysregulated miR-23b/27b expression in breast cancer is a crucial factor driving breast cancer progression (Hannafon et al. 2019).

Patients may get chemotherapy and/or targeted medicines depending on the subtype of their breast cancer, but in virtually all cases, intrinsic or acquired resistance will develop. The epithelial–mesenchymal transition (EMT), which occurs as tumor cells gain de novo or acquired treatment resistance, allows cancer cells to avoid the main tumor location and spread to other organs, ultimately causing the patient’s death. Thus, it is essential to find new targets that not only decrease tumor development but also make refractory cells more receptive to treatment and stop metastasis. Numerous oncogenic and tumor suppressor miRNAs have been linked to metastasis, treatment resistance, or cancer progression (Duan, et al. 2019; Fatimy et al. 2017a; Ahmad et al. 2014). Despite this, little information is available about miRNAs that can control tumor growth and EMT at the same time, acting as breast cancer therapy sensitizers and metastasis inhibitors. miR-644a has been identified as a potential inhibitor of breast cancer cell survival and inducer of epithelial–mesenchymal transition (EMT) by Raza et al. (2016). Additionally, it has been reported as a potential sensitizer for pleiotropic therapy. Research has shown that miR-644a is increased in bladder cancer, and its high expression has been associated with poorer overall survival in acute myeloid leukemia (AML). However, its expression has been shown to reduce the survival of prostate cancer cell lines and downregulate an isoform of the androgen receptor. Despite these findings, functional research on miR-644a is limited and there are no reported cases of its dysregulation in breast cancer (Duan, et al. 2019).

#### Ovarian cancer

The most lethal gynecologic malignancy is still ovarian cancer, despite advances in surgical procedures and chemotherapy approaches. This is primarily due to the late diagnosis of ovarian cancer as well as the appearance of resistance to conventional chemotherapy treatments such as platinum-based chemotherapy. Cisplatin, which effectively treats cancer of ovary and is also one among the most widely employed medications, produces cytotoxicity by interfering with apoptotic pathways to avoid or reduce medication resistance and improve the therapeutic effectiveness of ovarian cancer, it is critical to investigate novel therapeutic techniques and targets.

A number of miRNAs have reportedly been linked to medication resistance recently. By focusing on apoptosis and drug resistance genes, some miRNAs may change how responsive cancer cells are to anti-cancer medications. These unregulated miRNAs' effects on apoptosis and resistance provide up new therapy options for cancer. A family of proteins known as inhibitors of apoptosis (IAPs) plays a variety of biological roles, controlling cell migration, innate immunity, inflammation, and death. According to Plesner et al. (2005), the X-linked inhibitor of apoptosis protein (XIAP) serves as a highly effective regulator in mammalian cells. By directly interacting with Caspase-3, -7, and -9 in the apoptotic signaling Cascade, it can suppress Caspase activity. XIAP is a promising therapeutic target for treating cancer since it blocks a significant amount of the apoptotic process. According to reports, XIAP is upregulated in epithelial ovarian cancer, and this upregulation of XIAP may play a role in the emergence and development of ovarian cancer (Li et al. 2017; Eckelman et al. 2006).

It has been shown that miRNAs regulate the expression of tumor-associated genes in different cancer types. Therefore, they can serve as potential therapeutic targets. On chromosome 1p22, miR-137, which serves as a tumor suppressor by specifically targeting a variety of oncogenic mRNAs, is found. Since miR-137 is downregulated in a variety of tumor forms, it is possible to assume that it functions as a negative regulator of specific tumor-associated genes. For instance, miR-137 plays important roles in melanoma, glioblastoma, non-small cell lung cancer, colorectal cancer, ovarian cancer, and neuroblastoma. It also acts as a tumor suppressor by directly targeting carboxyl terminal-binding protein 1, which prevents melanoma cells from going through the epithelial–mesenchymal transition and triggers apoptosis. Among the targets of miR-137 in human ovarian cancer, only astrocyte elevated gene-1 (AEG-1) has been identified (Guo et al. 2013; Chen et al. 2018). Ovarian cancer cells depend on miR-137 for apoptosis control, and research by Li et al. (2017) found that miR-137 increased epithelial ovarian cancer cells to undergo cisplatin-induced apoptosis. This suggests that miR-137 is a promising potential therapeutic target. As a result, using therapy based on miR-137 may be advantageous for treating ovarian cancer clinically and overcoming drug resistance. Cell lines and tissues associated with ovarian cancer, the level of XIAP protein is inversely linked with the expression of miR-137 (Li et al. 2017).

Using a knockout cell model, Chen et al. (2018) investigated the role of miR-137 in ovarian cancer. Their findings showed that the miR-137-knockout cells had very little miR-137. MiR-137-knockout enhanced proliferation in A2780 cells, according to an MTT experiment. As a result, the CRISPR/Cas9 technology was effectively used to create the miR-137-knockout A2780 cell type, which demonstrated a practical impact (Chen et al. 2018).

#### Lung cancer

One of the main reasons for cancer-related deaths is lung cancer. More than 80% of lung cancer patients are non-small cell lung cancer (NSCLC), which has a current 5-year survival rate of only 15%. NSCLC survival rates have not improved much in recent decades despite advances in surgical resection, chemotherapy, and radiotherapy. As a result, new medicines and treatment targets are needed, and the development of early, secure, and noninvasive detection methods is vital for screening and diagnosis. Multiple cancers, including gastrointestinal cancer, breast cancer, prostate cancer, and lung cancer, have been linked to dysregulation of miRNAs. Numerous tumor-suppressive miRNAs influence medication resistance and signaling pathways connected to tumors. The identification of tumor-suppressive miRNAs that are crucially implicated in the emergence of lung cancer requires systematic screening. For in vivo systematic screening for tumor suppressor genes (TSGs), the CRISPR–Cas9 method is very effective. CRISPR–Cas9 with two gRNAs has been used to produce a substantially greater genomic loss in vitro for non-coding RNA knockout (Hong et al. 2020). Hong et al. (2020) discovered three tumor suppressors, including miR30b, miR146a, and miR-190b, and they demonstrated that miR-190b may have prevented the development of lung tumors by targeting the HUS1 gene. Previous studies have established the functional link between miR-146a and miR-190b and lung cancer, indicating that these two miRNAs might serve as diagnostic biomarker. Previous research has demonstrated a functional relationship between miR-146a and miR-190b and lung cancer, suggesting that these two miRNAs could be used as a biomarker for early diagnosis (Hong et al. 2020; Wang and Wang 2020; Gan 2018). Recent research suggests that a plasma microRNA panel of the genes miR-190b, miR-17, and miR-375 can distinguish between small cell and non-small cell lung cancers with excellent diagnostic accuracy (SCLC) (Lu et al. 2018; Patnaik, et al. 2012; Shaohua et al. 2018). According to the findings of the study by Hong et al. (2020), miR-190b is typically down-regulated in human lung cancer. Importantly, in vivo loss-of-function and gain-of-function research strongly suggests that miR-190b has a tumor-suppressive role in the development of lung tumors.

The RAD9–RAD1–HUS1 complex, which is loaded onto DNA under replicative stress and acts as a scaffold for the subsequent components that cause cell cycle arrest, contains the HUS1 checkpoint clamp component (HUS1). The RAD9–RAD1–HUS1 complex also plays a role in telomere maintenance and DNA damage repair. The development of tumors in the thyroid, prostate, breast, lung, and skin is frequently triggered by the aberrant expression of this complex component. According to in vitro research by Hong et al. (2020), HUS1 is miR-190b’s primary target, and HUS1 deletion in the KP model of lung cancer demonstrably slows the disease’s progression. This provides in vivo proof of HUS1’s critical function in the development of lung tumors. Furthermore, the inhibitory impact of miR-190b can be successfully overridden by ectopic production of HUS1 in vitro. Contrarily, antisense oligonucleotides that inhibit HUS1 increase the sensitivity of human lung carcinoma cells to the chemotherapy drug cisplatin, and high HUS1 expression is significantly associated with unfavorable clinicopathologic indicators in ovarian cancer. As a result, HUS1's potential function as a tumor promoter or a tumor suppressor may depend on the kind of malignancy and be organ-specific (Hong et al. 2020). The issue will also require further clarification in future.miR-421 was discovered and Q-PCR assays revealed that miR-421 overexpression is found in non-small cell lung cancer (NSCLC), particularly in A549 cells, in another work by Duan et al. (2019). Compared with healthy normal donors, blood samples from patients with lung cancer always contained more miR-421, indicating that miR-421 is a significant indicator of lung cancer. They demonstrated that inhibiting miR-421 expression with an antisense morpholino oligonucleotide (AMO) increased ROS levels and efficacy of treatment to paclitaxel in vitro and in vivo, and that high miR-421 expression may be responsible for at least some of the tolerance to paclitaxel in lung cancer patients. Their research revealed a novel function for miRNA-421 and the promising therapeutic effects of using AMO-421 to reduce paclitaxel sensitivity in NSCLC. Nanotags and novel AMO-421 formulations, namely those that bind to exosomes, are being developed to stabilize the RNA medication (Duan et al. 2019).

#### Glioblastoma (GBM)

The most frequent malignant brain tumor, glioblastoma (GBM), continues to be one of the worst diseases affecting people, with a survival time of just over a year, which has not changed over the previous 25 years. New molecular targets, ideas, and methods are desperately needed to treat this illness. Transcriptional factors and non-coding RNAs (such as miRNA), which are higher order switches that can affect a large number of downstream targets, are examples of regulatory molecules. Understanding how these switches work will further our knowledge and allow us to modify them for translatable therapies. Thus, a logical strategy to maximize this therapeutic potential is to understand the local regulators of such higher order modulators (for example, microRNA promoters). Numerous miRNAs have been shown to be predictive factors in glioma patient survival. Even though the delivery and stability of miRNA are currently ongoing research topics, they can be utilized to target whole circuits because each microRNA has several targets. Large microRNA clusters play an essential role in the biology of cancer, and this field of study is just getting started. Concentrating on miR-10b, a miRNA that is embedded inside the HOXD chromosomal locus and is linked to the growth, invasion, and metastasis of several cancers, including the GBM has been started after 10 years of studying in this area. Due to its distinct expression pattern, miR-10b is particularly significant in brain malignancies. While it is almost undetectable in healthy brain tissue, it becomes incredibly abundant in the majority of low- and high-grade gliomas of all subtypes as well as metastatic brain tumors (Fatimy et al. 2017a).

Patients with brain metastases from breast cancer have much greater levels of miR-10b than those with metastases in other organs. miR10b inhibition by chemically modified ASOs inhibits the growth, invasion, and metastasis of cultured glioma cells in aggressive cancer models (Ahmad et al. 2014). Despite being linked to cancer and having the potential to treat GBM, miR-10b's mechanism of action is still not fully understood. It is possible that miR-10b behaves differently than predicted by bioinformatics models since the predicted miR-10b targets were not enriched among the genes that were de-repressed by certain miR-10b inhibitors. The effects of miR-10b gene ablation on glioma growth in vitro and in vivo were examined in order to validate the specific loss of miR-10b function as a tumor-inhibiting therapy for GBM (Fatimy et al. 2017a; Ma et al. 2010). This potent method uses non-homologous end joining (NHEJ) to introduce insertions or deletions at precise locations in the mammalian genome, allowing for targeted DNA double-strand breaks (indels). It has been shown that miR-10b gene editing is detrimental to all glioma cells and GBM-initiating stem cells (GSCs) because miR-10b expression is essential for the survival of both types of cells. Additionally, they demonstrated that targeted miR-10b gene editing through lentivirus-mediated CRISPR–Cas9 inhibits the growth of orthotopic GBM in mice, indicating this method as a viable treatment for GBM (Fatimy et al. 2017a).

As a result, the tumor-specific characteristic of glioma addiction to miR-10b appears to be linked to the gene’s de-repression in the brain microenvironment, where it is typically repressed. Additionally, the distinct onco-miR dependency of GBM raises the possibility that the tumor could be eliminated by focusing on a single miRNA gene. Synthetic miR-10b inhibitors were administered, and the results on aggressive GSC-based models of orthotopic GBM were significant but short-lived. This was most likely caused by the ASOs' poor distribution and absorption in intracranial GBM as well as the drug's dilution in the tumor's active growth. An alternate approach that could improve the effectiveness of tumor cell eradication and do away with the requirement for ongoing delivery of miR-10b inhibitors to intracranial brain tumors is gene editing, which is based on irreversible miR10b inactivation (Fatimy et al. 2017a; Teplyuk et al. 2016). Accordance with the outcome from a study by El Fatimy et al., miR-10b ASO inhibitors and CRISPR–Cas9 plasmid- and virus-mediated miR-10b targeting have no effect on the survivability of mouse primary astrocytes or neurons or the locus editing of normal brain cells (Fatimy et al. 2017b).

#### Pancreatic ductal adenocarcinoma (PDAC)

Aggressive pancreatic ductal adenocarcinoma (PDAC) is a malignancy with poor prognoses and few effective treatments. Therefore, it is crucial to understand the signaling pathways involved in the etiology of PDAC in order to find new possible therapeutic gene targets. PDAC was discovered to have significantly lower levels of the FOXA2 transcription factor than control pancreatic tissues. Functional studies demonstrated that FOXA2 inhibits pancreatic cancer cell proliferation, migration, invasion, and colony formation to serve as a tumor suppressor. Pancreatic cancer has been linked to a number of important oncogenic miRNAs, such as miR-483-3p, miR-155, miR-21/miR-221, miR-27a, miR371-5p, and miR-21/miR-23a/miR-27a. Pancreatic oncogenesis' functional characteristics are decreased by inhibition of oncogenic miRNAs (Vorvis et al. 2016). By analyzing short RNAs in human embryonic stem cells, the miR-1246 was discovered in 2008 (Morin et al. 2008). miR-1246’s gene, *MIR1246*, was located on human chromosome 2q31.1, and p53 was identified to control the expression of the gene. MiR-1246 has been discovered to function as an oncomiR in the setting of cancer, promoting tumor angiogenesis, growth and metastasis, migration and invasion, and cancer stemness in a variety of malignancies, including PDAC. PDAC exosomes are substantially enriched in miR-1246, and patients with localized PDAC had higher levels of miR-1246 and miR-196a in their plasma. These earlier results highlight the biological importance of miR-1246 in human cancer and highlight the necessity for further research into this miRNA species’ role in cancer biology (Xu et al. 2019).

Over the past 10 years, research has shown that various human malignancies have changed miR-199a expression. The expression of miRNA-199 is specifically elevated in ovarian cancer cells and cervical carcinomas. In particular, miR-199a oncogenic qualities in pancreatic cancer were shown by an increase in pancreatic cell proliferation, migration, and invasion in vitro when miR-199a was overexpressed in pancreatic cancer cells. In 2016, Vorvis et al. identified a new signaling pathway involved in pancreatic oncogenesis that includes the miR-199a tumor suppressor gene and FOXA2 tumor suppressor gene (Vorvis et al. 2016).

#### Renal cell carcinoma (RCC)

RCC, renal cell carcinoma, a malignant cancer of the kidney's tubular cells, has a clear cell subtype that makes up about 80% of Cases (ccRCC). Due to recurrence or distant metastasis, the 5-year survival rate for advanced-stage RCCs is dismal (5–10%), and almost 30% of RCCs are found to have metastasized malignancies. For patients with metastatic or recurrent RCC, molecularly targeted therapies such as multi-targeted receptor tyrosine kinase (RTK) or mTOR inhibitors are frequently employed as RCC therapy nowadays. These therapies do not, however, treat cancer; they just slow its progression. In order to find new therapeutic targets, there is therefore more motivation to explain the molecular mechanisms behind RCC growth and progression. Previous research revealed that the most elevated miRNAs in clear cell renal cell carcinoma were miR-885-5p, miR-1274, 210-3p, 224, and miR-1290 (Yoshino et al. 2017).

According to McCormick et al. (2013), clinical ccRCC tissues have usually higher levels of miR-210-3p than normal tissues. The expression of miR210-3p was found to be decreased in high-grade and late-stage clear cell renal cell carcinoma (ccRCC) compared to low-grade and early-stage ccRCC. Furthermore, higher expression of Ki-67, a marker of advanced cancer and poor survival, was observed in cases where miR210-3p expression was decreased.

Through the regulation of EMT, TWIST1 is known to play significant roles in various physiological processes related to invasion and metastasis. TWIST1 has also been demonstrated to play a role in preventing apoptosis (Puisieux et al. 2006; Endo et al. 2019). Compared to low TWIST1 and high miR-210-3p expression, high TWIST1 and low miR-210-3p expression are linked to worse overall and disease-free survival. These results imply that miR-210-3p-mediated TWIST1 repression promotes the advancement of renal cell carcinoma (Yoshino et al. 2017).

#### Biliary tract cancers

A growing number of people are developing biliary tract malignancies (BTCs), which include cholangiocarcinoma (CCA) and gallbladder cancer. The need for a deeper understanding of BTC biology and mechanisms of responsiveness to treatment is highlighted by the lack of viable radical treatments and the quick failure of palliative ones. Eighty percent of BTC patients come at an advanced stage when only chemotherapy with cisplatin and gemcitabine is an option for treatment (CG) (Valle et al. 2010). While initial resistance is found in 20% of individuals, only 11% of patients benefit long-term from chemotherapy. After an initial response or illness stabilization, the majority of patients develop secondary resistance, which is the cause of a median overall survival of fewer than 12 months globally (Marin et al. 2016). Chemotherapy-induced cellular inhibition or death may be prevented through the synergistic action of multiple chemo-resistance mechanisms. The acquisition of stem cell characteristics, a feature observed across various types of tumors, has been identified as a common driver of resistance (Marin et al. 2016; Huang et al. 2019). Recently, the field of miRNA-based treatments has grown and moved into the phase of clinical research. It is desirable for miRNAs to target several pathways since doing so may delay the emergence of compensatory pathways.

Data from the MesomiR1 study have demonstrated the viability of a treatment strategy based on the replacement of miRNA in patients with human cancer. This strategy has two significant drawbacks, though: (1) toxicity associated with the immuno-stimulatory effects of delivery methods that are encapsulated, and (2) off-target effects brought on by an abnormally high quantity of miRNA in the cellular system (Zandwijk et al. 2017). Instead of introducing a disturbance that alters cellular homeostasis, a method based on the suppression of miRNAs would lower the possibility of off-target effects by altering the physiological level of a miRNA. Additionally, it is possible to hypothesize that this process is also shared by other cancer types, which may call for research in other solid tumors (Carotenuto et al. 2020).

## Conclusion

The ability of CRISPR–Cas9 to specifically modify genomes has ushered in a new phase in human evolution. We anticipate a new era in the treatment of several ailments, including cancer. This cutting-edge gene editing technology has made it possible to take a modern approach to study the part that various signaling molecules play in the development of cancer.

Consequently, it will help us implement special tactics to create fresh cancer treatments. The CRISPR–Cas9 technique's versatility in programming has highlighted its rise as a modeling tool for cancer scientists. By offering creative approaches for customized medicine, gene therapy, immunotherapy, and the treatment of genetic disorders, CRISPR–Cas9-based technology has the ability to restructure the notions in cancer biology. In future, we prospect this technology will provide an essential genome array across almost all cancer cell lines. Although CRISPR–Cas is a powerful genome editing tool, it also faces many obstacles, including ethical issues. One major ethical concern is the potential for creating “designer babies”, where the genetic makeup of a fetus is altered to produce certain desired characteristics. This raises questions about the morality of such actions and the potential for eugenics. Another concern is the possibility of unintended consequences, such as the emergence of antibiotic-resistant bacteria because of using CRISPR–Cas to edit bacterial genomes. The CRISPR–Cas technology is relatively new, and its cost of use is relatively high, which may limit its accessibility in low-income countries. This has raised concerns about a “genetic divide”, where access to the technology is limited to certain individuals or countries. Additionally, the high cost of the technology may also limit its potential use for certain research purposes in low-income countries.

## Data Availability

There is no data obtained for this review paper.
